# Harnessing Metallic Nanoparticle-Based Anodes for Red-Shifting and Reshaping Electroluminescence toward the Near-Infrared Region

**DOI:** 10.1021/acsami.5c08869

**Published:** 2025-07-07

**Authors:** Nurul Ridho Al Amin, Ming-Jun Lin, Jui-Ming Wang, Zu-Po Yang, Hai-Ching Su, Chih-Hao Chang

**Affiliations:** † Department of Electrical Engineering, 34895 Yuan Ze University, Taoyuan 32003, Taiwan; ‡ Institute of Photonic System, 529897 National Yang Ming Chiao Tung University, Tainan 71150, Taiwan; § Institute of Lighting and Energy Photonics, National Yang Ming Chiao Tung University, Tainan 71150, Taiwan

**Keywords:** localized surface plasmon resonance, color-tunable, silver nanoparticles, gold nanoparticles, organic light-emitting diodes

## Abstract

Leveraging conventional red-emitting materials, a color-tuning strategy is used to develop high-performance near-infrared (NIR) organic light-emitting diodes (OLEDs). This study presents a practical approach for achieving color-tunable optically impaired OLEDs through localized surface plasmon resonance (LSPR), which extends the emission of red-emitting materials into the NIR region, thereby eliminating the need for dedicated NIR emitters. The method utilizes silver (Ag) and gold (Au) nanoparticle-based anodes modified with titanium dioxide (TiO_2_) coatings under various rapid thermal annealing (RTA) treatments (200 °C, 400 °C, and 600 °C). Three TiO_2_ coating techniques—overcoating, stacking, and sandwiching—were compared in terms of their influence on the LSPR behavior and electroluminescence (EL) spectra. UV–vis spectroscopy, including transmittance and absorbance analyses, confirmed that the different coating methods facilitated LSPR formation and thus modulated EL characteristics and induced distinct spectral features. Ag nanoparticles exhibited stronger LSPR responses and greater color-tunability than Au nanoparticles. Scanning electron microscopy revealed that RTA promoted island-like nanoparticle growth and minimized agglomeration, while TiO_2_ coatings enhanced island formation, resulting in well-defined and narrower absorbance peaks. The overcoating approach successfully red-shifted the primary emission peak of red phosphorescent OLEDs from 663 to 723 nm, achieving a maximum external quantum efficiency of 12.91% and a low turn-on voltage (*V*_on_) of 2.41 V. Furthermore, a broad EL emission spectrum (full width at half maximum = 199 nm) spanning 643–842 nm was achieved using the TiO_2_ stacking configuration. These findings highlight the potential of Ag nanoparticle-based anodes for color-tunable NIR OLEDs and introduce a stacking coating technique with potential for broad-wavelength applications.

## Graphical abstract

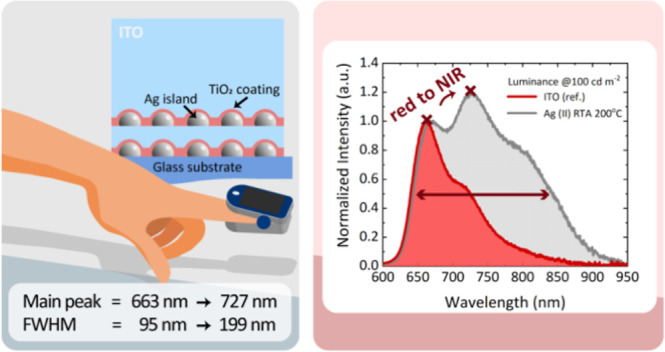

## 1 Introduction

Near-infrared (NIR) organic light-emitting diodes (OLEDs) are highly promising for applications in medical diagnostics, photodynamic therapy, bioimaging, and night vision, owing to their deep tissue penetration and minimal interference from ambient visible light.^1–3^ However, their development is constrained by a lack of stable NIR emitters and the need for optimized device architectures to improve light extraction, reduce energy loss, and maintain the charge balance. One major challenge lies in the inherently narrow energy bandgaps of NIR emitters, which increases susceptibility to nonradiative decay, leading to reduced efficiency and operational stability. Consequently, a significant portion of energy is dissipated as heat rather than converted into light, reducing device efficiency and longevity.^4–8^ To overcome these challenges, an alternative device architecture using existing red emitter materials is needed as a practical approach to achieve high-performance NIR OLEDs.

Among various optimization strategies proposed for NIR OLEDs, color-tunable approaches that leverage techniques widely used in sensing applications have shown promise, particularly localized surface plasmon resonance (LSPR).^9,10^ LSPR was initially used to enhance OLED performance through the strong coupling effect between emitted light and metallic nanoparticles (NPs).^11–14^ Lee et al. integrated LSPR formation into OLED architectures for color-tunable applications.^15^ The LSPR effect arises from the collective oscillation of conduction electrons on metallic NPs under incident light, enabling enhanced light absorption and scattering across visible and NIR wavelengths. Unlike surface plasmon resonance (SPR), which occurs at planar metal–dielectric interfaces, LSPR is a localized effect that originates when the NP dimensions are smaller than the excitation wavelength. This effect has been exploited to enhance light extraction and induce wavelength shifts by modifying the oscillation frequency.

During deposition, metallic NPs typically exhibit Volmer–Weber growth, forming three-dimensional (3D) islands that expand and coalesce into a semicontinuous or conductive film.^16^ These islands have distinct boundaries and support localized free electron oscillations when excited by incident light, which are essential in LSPR.^17^ Various strategies have been explored to precisely tune LSPR characteristics and achieve the desired optical properties.^18^ LSPR formation is susceptible to material composition, size, morphology, and surrounding environment.^19–21^ Silver (Ag), aluminum (Al), gold (Au), and copper (Cu) are commonly used materials to induce the LSPR formation. Moreover, composite materials such as Au/Ag can facilitate LSPR formation, while adjusting their composition ratio allows tuning of the LSPR characteristics.^22,23^ NP size is a critical factor for balancing light scattering and absorbance characteristics. For instance, Ag and Au NPs under 50 nm mainly exhibit stronger light absorption, while larger ones favor scattering, causing spectral broadening and wavelength shifting.^24–27^ NP morphology also significantly affects LSPR characteristics, with Chen et al. demonstrating that Au NPs exhibit tunable plasmon wavelengths from 527 nm (spheres) to 1141 nm (branches) and 645–1096 nm (bipyramids).^28^ Precise control over NP morphology and size distribution is crucial because slight variations can significantly alter the absorbance spectra.^29,30^ For instance, small changes in Ag nanobar length, while maintaining constant width and height, can shift the absorbance spectra from the visible to the NIR range.^31^ However, nonspherical NPs exhibit uneven surface plasmon distributions, leading to multiple absorbance peaks that are unsuitable for applications requiring a single well-defined wavelength.^32^

Rather than altering the size or shape of NPs, scalable LSPR tuning can also be achieved by modifying the surrounding dielectric environment.^33^ Metal oxide coatings are commonly used to tune LSPR characteristics, as they influence the absorbance spectra based on the material selection, thickness, and deposition method. Various metal oxide coating materials, including SiO_2_, TiO_2_, SnO_2_, ZrO_2_, and Al_2_O_3_, have been used to alter the metallic NP environment.^34–37^ In particular, dielectric coatings such as TiO_2_ can red-shift the resonance wavelength and narrow the absorbance spectrum due to their high refractive index and favorable plasmon–dielectric interactions.^38^ Studies have shown that coating Ag NP islands using a dielectric medium of TiO_2_ coating can significantly tune the LSPR wavelength from 482 to 1022 nm via overcoating and up to 1310 nm using the sandwiching technique.^39^

This study presents a practical approach for achieving color-tunable OLEDs through LSPR, which extends the emission of red-emitting materials into the NIR region, thereby avoiding the need for dedicated NIR emitters. The method utilizes Ag and Au NP-based anodes modified with TiO_2_ coatings subjected to various rapid thermal annealing (RTA) treatments. The Ag and Au NPs were selected due to their strong LSPR response and relatively low optical loss. The LSPR peaks of spherical-shaped NPs typically occur within 380–450 nm for Ag and 510–580 nm for Au, while the photoluminescence (PL) spectra of red emitters fall within 580–700 nm. By incorporating an LSPR that absorbs light in the 400–600 nm range, the electroluminescence (EL) spectra can be red-shifted toward the NIR region. Here, the LSPR structure was investigated using a metallic NP-based anode with three different TiO_2_ coating techniques under various RTA treatments (200 °C, 400 °C, and 600 °C): (1) overcoating: TiO_2_ is applied to the metallic NPs after their deposition, (2) stacking: the overcoating process is performed twice, with TiO_2_ coated after each NP deposition, and (3) sandwiching: metallic NPs are coated with TiO_2_ both before and after deposition.

UV–vis spectroscopy using transmittance and absorbance analysis data of various TiO_2_ coating techniques exhibited distinct profiles, confirming that the designed NP layers facilitate LSPR formation and influence EL characteristics. Ag NPs demonstrated a stronger LSPR response, which is more favorable for color-tunable applications compared to that of Au NPs. Scanning electron microscopy (SEM) results revealed that RTA treatment promotes NP island growth and reduces NP agglomeration, while TiO_2_ coating improves 3D island formation, producing well-defined and narrow absorbance spectra. This study successfully shifted the primary emission peak of red phosphorescent OLEDs from 663 to 723 nm while achieving excellent EL performance with a maximum external quantum efficiency (EQE) of 12.91% and a low turn-on voltage (*V*_on_) of 2.41 V. These results are comparable to those achieved with a reference device with a max EQE of 13.73% and *V*_on_ of 2.42 V. Additionally, a broad EL emission with a full width at half maximum (FWHM) of 199 nm was achieved using the TiO_2_ stacking technique covering a wavelength range of 643–842 nm. OLEDs with a broad spectrum are suitable for various phototherapy lamp sources, which helps to improve absorption efficiency.^40^ These findings highlight the potential of Ag NP-based anodes for color-tunable NIR OLEDs and introduce a stacking coating technique with potential for broad-wavelength applications.

## 2 Results and Discussion

### 2.1 Preparation and Characterization of Metallic Nanoparticles

Metallic NPs can be synthesized by chemical or physical methods. While chemical synthesis offers simplicity, scalability, and high yield, it typically involves the use of hazardous solvents that pose environmental and health risks. In contrast, physical deposition techniques such as sputtering and thermal evaporation offer solvent-free processing, rapid deposition, and precise control over film thickness and morphology, albeit at a higher cost.^41^ In this study, metallic NPs were synthesized by using a physical deposition method. Due to equipment limitations, Ag NPs were produced via sputtering, while Au NPs were fabricated through thermal evaporation. Both deposition processes were conducted in a vacuum chamber to ensure optimal conditions.

UV–vis spectroscopy was used to characterize LSPR behavior, with absorbance spectra indicating resonance peak positions and transmittance data revealing optical transparency.^42^ Ag and Au NP films with thicknesses of 3, 5, and 7 nm were deposited on glass substrates without RTA treatment to evaluate the LSPR formation in bare films. As shown in Figure S1, LSPR formation was confirmed but was accompanied by broad absorption peaks and limited transmittance (<80%). Sheet resistance measurements (Table S1) indicate that increased film thickness improved conductivity but at the expense of optical transparency, highlighting the need for balance in OLED applications to optimize light outcoupling. The absorbance spectral differences depend on NP thickness, consistent with results from previous studies.^43,44^ To further enhance LSPR formation, TiO_2_ coatings of varying thicknesses (0, 7, 10, and 13 nm) were applied to Ag NP films subjected to RTA at 100 and 400 °C. RTA is known to promote surface diffusion and island growth in NP films.^45,46^ As shown in Figure S2, higher annealing temperatures of TiO_2_-coated films led to stronger and more defined LSPR features, with optimal absorption achieved with a 10 nm TiO_2_ layer. At 100 °C RTA, broad absorbance spectra indicate incomplete Ag island formation, whereas 400 °C RTA results in well-defined LSPR and narrower absorbance, suggesting the formation of isolated Ag islands.

The influence of RTA treatments and TiO_2_ coatings on the NP morphology is further demonstrated in Figure 1. To ensure a clear comparison, optimized parameters were used to analyze Ag and Au NP films under different RTA treatments (200 °C, 400 °C, and 600 °C). Both Ag and Au NP films showed high NIR transmittance regardless of RTA condition, making them suitable for NIR applications. For uncoated films, LSPR peaks appeared at 419–456 nm (Ag) and 529–550 nm (Au), while TiO_2_-coated films exhibited red-shifted peaks at 526–574 nm (Ag) and 603–648 nm (Au), confirming the dielectric tuning effect. LSPR occurs when metallic NPs interact with incident light at specific wavelengths, resulting in the collective oscillation of localized free electrons on the NP surface. Effective LSPR formation requires isolated 3D NP islands, as excessive aggregation broadens the resonance peak and reduces plasmonic intensity.^39^ In isolated NP islands, each particle resonates independently and produces a high-intensity LSPR peak with well-defined and narrower characteristics. In contrast, agglomerated NPs facilitate plasmon coupling between neighboring particles, leading to peak shifting and broader, weaker absorbance peaks with increased scattering losses. RTA facilitates island separation, while TiO_2_ coatings assist in refining their shapes and distribution. The blueshift phenomenon in the absorbance peak can be attributed to several factors, including variation in NP size or modification in the optical properties of the surrounding medium.^47–49^ In our case, the absorbance peak not only shifts toward shorter wavelengths (blueshift) but also becomes narrower, as indicated by a reduced FWHM. This behavior suggests enhanced electron localization due to reduced interparticle coupling resulting from thermal treatment. This phenomenon can be verified by analyzing the NP film surface morphology.

**Figure 1:**
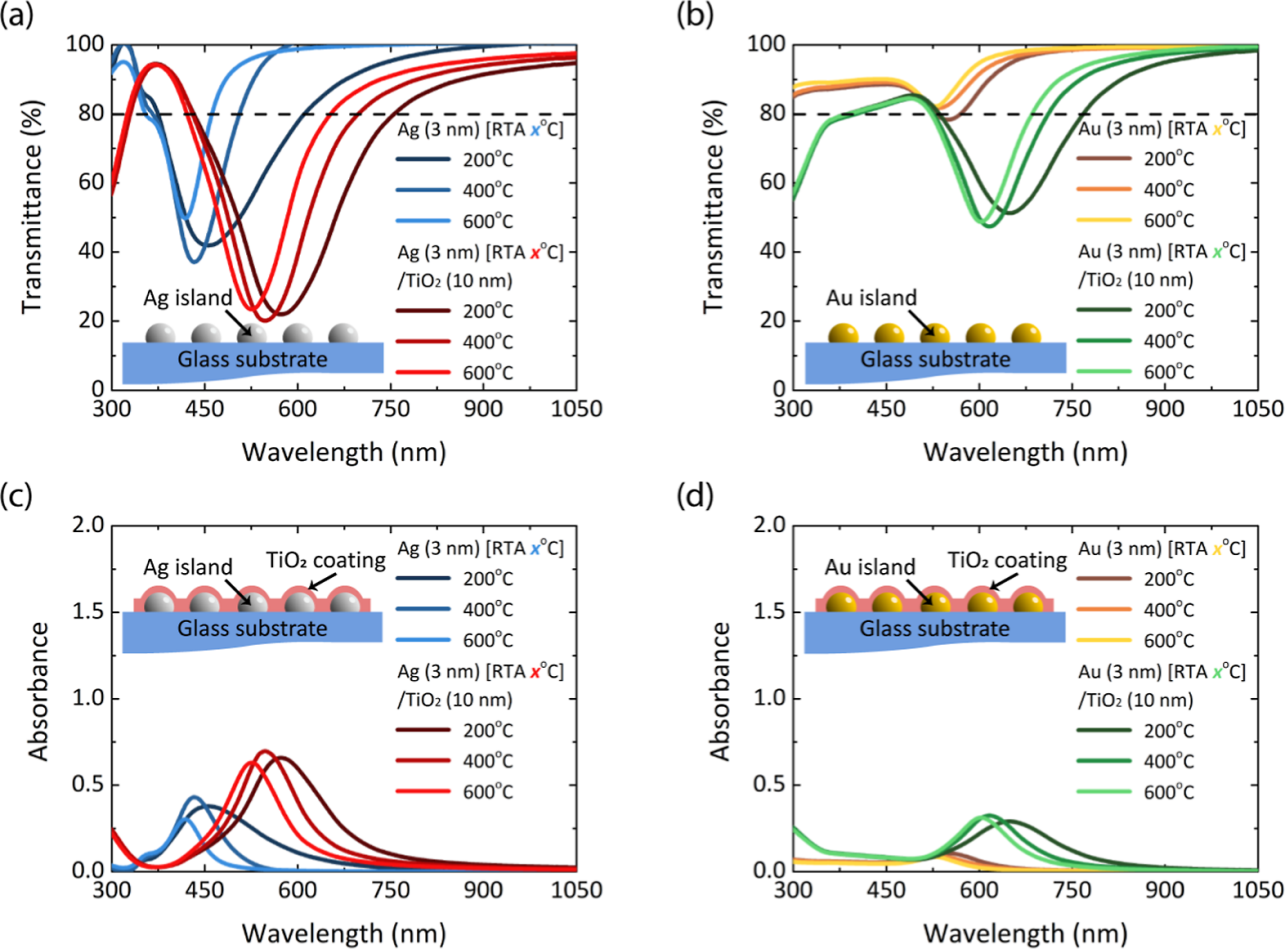
Optical transmittance and absorbance of various metallic nanoparticle thin films under different RTA treatments for (a,c) Ag; Ag/TiO_2_, and (b,d) Au; Au/TiO_2_.

SEM is an effective method for visualizing surface morphology and analyzing NP size and distribution in films.^50^ The surface morphology of the metallic NP films was investigated to identify changes under different RTA treatments, both with and without a TiO_2_ coating. SEM morphologies of Ag NPs under different RTA treatments are shown in Figure 2, while those of Au NPs are presented in Figure S3. The results for both Ag and Au NP films indicate that higher annealing temperatures promote 3D island growth and the formation of larger metal clusters while reducing island agglomeration and boundary defects.^51–53^ Additionally, TiO_2_ coatings exhibit a pronounced annealing effect that influences their crystalline structure, which depends on temperature through distinct profiles: amorphous, anatase, and rutile.^54–56^ TiO_2_ exhibits an amorphous phase at lower annealing temperatures (<200 °C), forming loosely packed agglomerates due to weak interparticle forces. At higher annealing temperatures (>750 °C), TiO_2_ transforms into the rutile phase, promoting grain growth and causing significant particle aggregation and coalescence. The anatase phase (200–700 °C) supports controlled NP growth and provides suitable conditions for the emergence of LSPR formation.^57^

**Figure 2:**
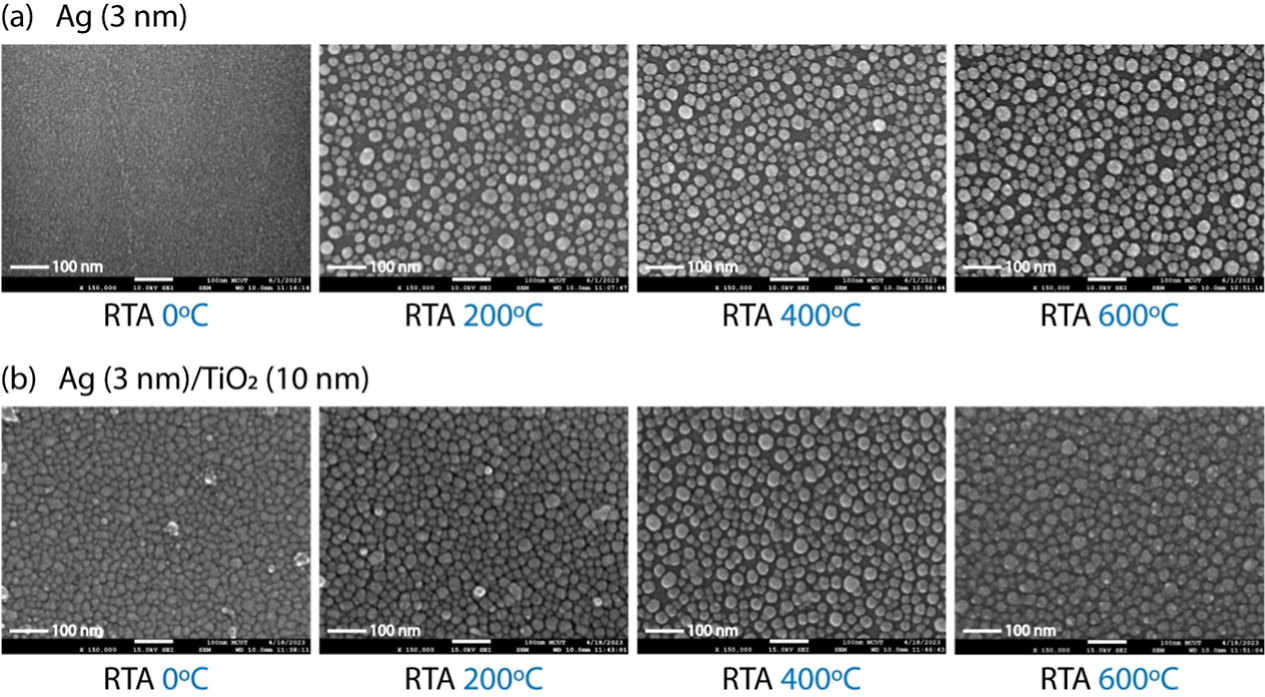
SEM images of Ag nanoparticle thin films under different RTA treatments for (a) Ag and (b) Ag/TiO_2_.

Significant differences between annealed and nonannealed films highlight the importance of isolated 3D island formation for LSPR. Without RTA (0 °C), both coated and uncoated Ag and Au NP films exhibit agglomerated island formation and contain smaller NP sizes, consistent with broad absorbance spectra. According to Hutter and Fendler, a broad absorbance spectrum occurs when the metallic NPs are significantly smaller than the incident wavelength.^19^ This trend aligns with absorbance spectra, confirming that increasing the annealing temperature enhances 3D island formation, producing well-defined and narrow absorption spectra. TiO_2_ coating further promotes this process and strengthens LSPR formation. These findings provide direct evidence of the synergistic effect of thermal annealing and dielectric coating in promoting well-defined LSPR-active nanostructures.

To investigate the influence of various TiO_2_ coating techniques under different RTA treatments (200 °C, 400 °C, and 600 °C) on LSPR characteristics, we introduce three manners of metallic NP-based anode structure: overcoating, stacking, and sandwiching coating. The configurations for metallic NP-based anode coating are as follows: (1) overcoating: TiO_2_ is applied to the metallic NPs after deposition in a structure as follows: Ag (I) or Au (I): Glass/Ag or Au (3 nm)/TiO_2_ (10 nm)/indium thin oxide (ITO) (120 nm). (2) Stacking: the overcoating process is performed twice, with each NP deposition followed by coating with TiO_2_ in a structure as follows: Ag (II) or Au (II): Glass/Ag or Au (3 nm)/TiO_2_ (10 nm)/ITO (30 nm)/Ag or Au (3 nm)/TiO_2_ (10 nm)/ITO (80 nm). (3) Sandwiching: metallic NPs are coated with TiO_2_ both before and after deposition in a structure as follows: Ag (III) or Au (III): Glass/TiO_2_ (10 nm)/Ag or Au (3 nm)/TiO_2_ (10 nm)/ITO (110 nm). The three TiO_2_ coating techniques (overcoating, stacking, and sandwiching) differ in how they modify the dielectric environment around the metallic NPs, which directly affects LSPR behavior.^39^ In the overcoating technique, TiO_2_ is deposited on top of metallic NPs, while the bottom remains in contact with the glass substrate. This partial dielectric coverage creates an asymmetrical environment, resulting in a moderate red-shift due to limited refractive index change. The stacking method involves two overcoating steps, where one metallic NP layer is placed on the ITO and another on the glass substrate. This also forms an asymmetrical dielectric environment but yields a stronger absorption profile due to a higher NP density. The sandwiching, by contrast, wraps the metallic NPs between two TiO_2_ layers, creating a symmetrical dielectric environment. This full coverage leads to the most enormous LSPR redshift, as the surrounding refractive index is increased uniformly.

UV–vis spectroscopy, including transmittance and absorbance measurements, was used to analyze the LSPR characteristics. The results for the Ag NP-based anode are presented in Figure 3, while those for the Au NP-based anode are shown in Figure S4. Ag NPs exhibited more pronounced LSPR behavior compared to their Au counterparts. This was expected since the results follow the trend shown in Figure 1, where Ag NPs exhibited stronger LSPR characteristics than Au NPs. The effect of RTA treatment on enhanced 3D island formation showed a clear trend on both Ag and Au NP thin films, where higher annealing temperatures led to well-defined and narrower absorbance spectra. Generally, transparent conductive electrodes, such as ITO, require a transmittance value over 80% for optimal performance.^58,59^ Similarly, the overcoating technique for Ag and Au NP thin films achieved transmittance rates exceeding 80% in the NIR region, making them suitable for NIR applications. The stacking configuration showed higher absorbance and lower transmittance due to the doubling of the Ag LSPR layer, which increased the light absorption. The sandwiching technique displayed broader absorbance spectra, as the full coverage of TiO_2_ enhanced absorbance properties and induced more island agglomeration formation.

**Figure 3:**
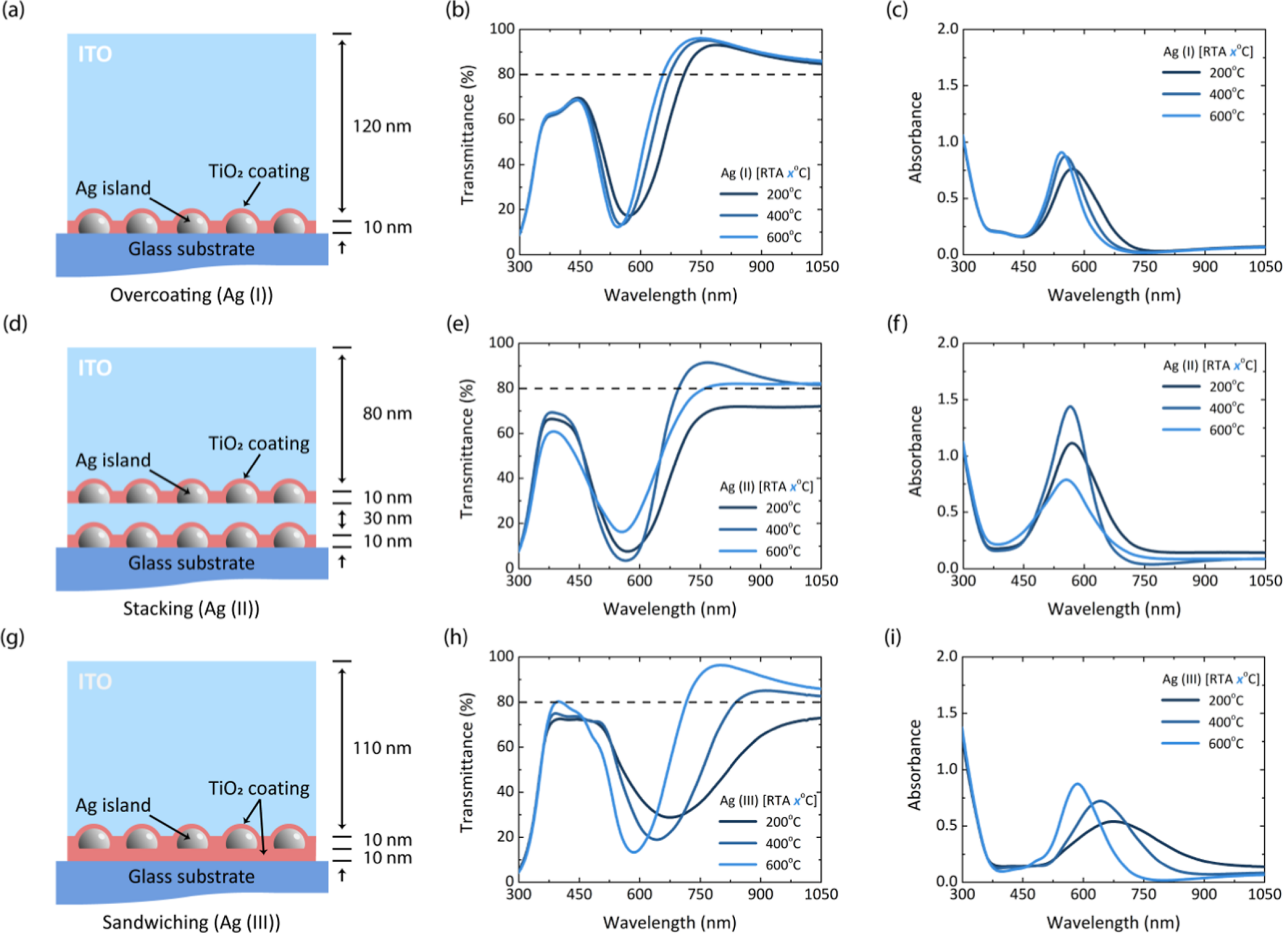
Thin film layer of various TiO_2_ coating techniques using Ag nanoparticles deposited on glass substrate for (a) overcoating (Ag (I)): Ag/TiO_2_, (d) stacking (Ag (II)): Ag/TiO_2_/ITO/Ag/TiO_2_, and (g) sandwiching (Ag (III)): TiO_2_/Ag/TiO_2_. The optical transmittance and absorbance of various TiO_2_ coating techniques under different RTA treatments for the thin films (b,c) Ag (I), (e,f) Ag (II), and (h,i) Ag (III).

To investigate the electrical properties of the metallic NP-based anode using various TiO_2_ coating techniques under different RTA treatments, carrier concentration (*n*), mobility (μ), resistivity (ρ), and sheet resistance (*R*_s_) were measured and are summarized in Table 1 for Ag NPs and Table S1 for Au NPs. The electrical behavior of Ag and Au NPs exhibited minimal variation across the different RTA treatments, suggesting that annealing has a limited impact on their electrical properties. Similarly, changing the coating techniques resulted in no significant differences or consistent trends across different configurations. This inconsistency may be attributed to defect disorder on TiO_2_, which could trap charges or photons within the film at unpredictable concentrations.^60–62^ Considering that the reference ITO data are based on a pure 130 nm ITO anode and the overcoating, stacking, and sandwiching anodes primarily comprise ITO, their electrical properties are expected to be comparable. Therefore, it can be concluded that LSPR mainly influences the optical properties rather than the electrical characteristics of the anode.

**Table 1:** Electrical Properties of the Ag Nanoparticle-Based Anode Using Various TiO_2_ Coating Techniques under Different RTA Treatments for the Thin Films Ag (I), Ag (II), and Ag (III)^a^

	TiO_2_ coating techniques								
thin film	overcoating Ag (I)			stacking Ag (II)			sandwiching Ag (III)		
structure	glass/Ag (3 nm)/TiO_2_ (10 nm)/ITO (120 nm)			glass/Ag (3 nm)/TiO_2_ (10 nm)/ITO (30 nm)/Ag (3 nm)/TiO_2_ (10 nm)/ITO (80 nm)			glass/TiO_2_ (10 nm)/Ag (3 nm)/TiO_2_ (10 nm)/ITO (110 nm)		
Ag NPs RTA [*x* °C]	200	400	600	200	400	600	200	400	600
*n* [×10^21^ cm^–3^]	1.2	1.8	2.1	0.7	3.0	2.0	3.7	0.8	0.8
μ [cm^2^ V^–1^ s^–1^]	9.1	9.1	5.0	15.0	15.7	15.3	12.1	19.5	16.1
ρ [×10^–4^ Ω cm]	5.9	6.1	6.2	5.0	3.3	3.4	4.9	4.9	5.3
*R*_s_ [Ω sq^–1^]	45.1	46.7	47.5	38.7	25.5	25.8	37.9	37.8	41.0

a *n*: carrier concentration, μ: mobility, ρ: resistivity, *R*_s_: sheet resistance.

### 2.2 OLED Device Fabrication and Characterization

In this section, a red phosphorescent OLED was fabricated to evaluate the impact of LSPR formation for color-tunable applications using various TiO_2_ coating techniques and RTA treatments (200 °C, 400 °C, and 600 °C). The device architectures and material structures are illustrated in Figure 4. The OLED structure comprises: anode (ITO or metallic NP-based anode (types I–III)) (130 nm)/HAT-CN (10 nm)/TAPC (40 nm)/TCTA (5 nm)/TCTA: CN-T2T: Ir­(fliq)_2_(acac) (1:1:5 wt %, 25 nm)/CN-T2T (50 nm)/lithium fluoride (LiF) (1.2 nm)/Al (120 nm). Ag and Au NP-based anodes are evaluated for their EL performance, where a pure ITO anode serves as a reference device. The TiO_2_ coating variations are classified as follows: type I (overcoating, including Ag (I) and Au (I)), type II (stacking, including Ag (II) and Au (II)), and type III (sandwiching, including Ag (III) and Au (III)). In this configuration, HAT-CN serves as the hole injection layer; TAPC and TCTA as hole transport layers; TCTA:CN-T2T as the exciplex host for the red emitter; CN-T2T as the electron transport layer; LiF as the electron injection layer; and Al as the cathode. The TCTA: CN-T2T exciplex host is known for its efficient exciplex formation and is commonly used in red OLED systems.^63–65^ The red phosphorescent emitter, Ir­(fliq)_2_(acac), exhibits a peak emission between 650 and 670 nm.^66^

**Figure 4:**
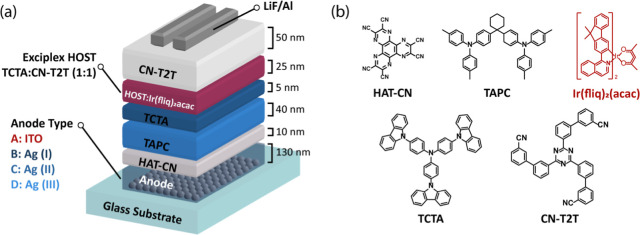
(a) OLED device architecture and (b) material structures of used materials.

To evaluate the effects of various TiO_2_ coating techniques under different RTA treatments, the EL performance data, including normalized EL spectra, luminance–voltage–current density (*L*–*V*–*J*) characteristics, EQE, current efficiency, and power efficiency, were analyzed. Figures 5 and S5 emphasize the importance of absorption spectra to tune the desired LSPR characteristics. Each coating technique presents distinct absorption spectra and overlaps with the Ir­(fliq)_2_(acac) emission spectrum, providing clear evidence of the LSPR-induced spectral alteration. However, because the absorption spectra do not fully cover the emitter spectrum, the LSPR absorbance shape still retains the original emitter emission shape. The primary differences observed are the EL peak positions and FWHM, demonstrating apparent LSPR-induced spectral reshaping.

**Figure 5:**
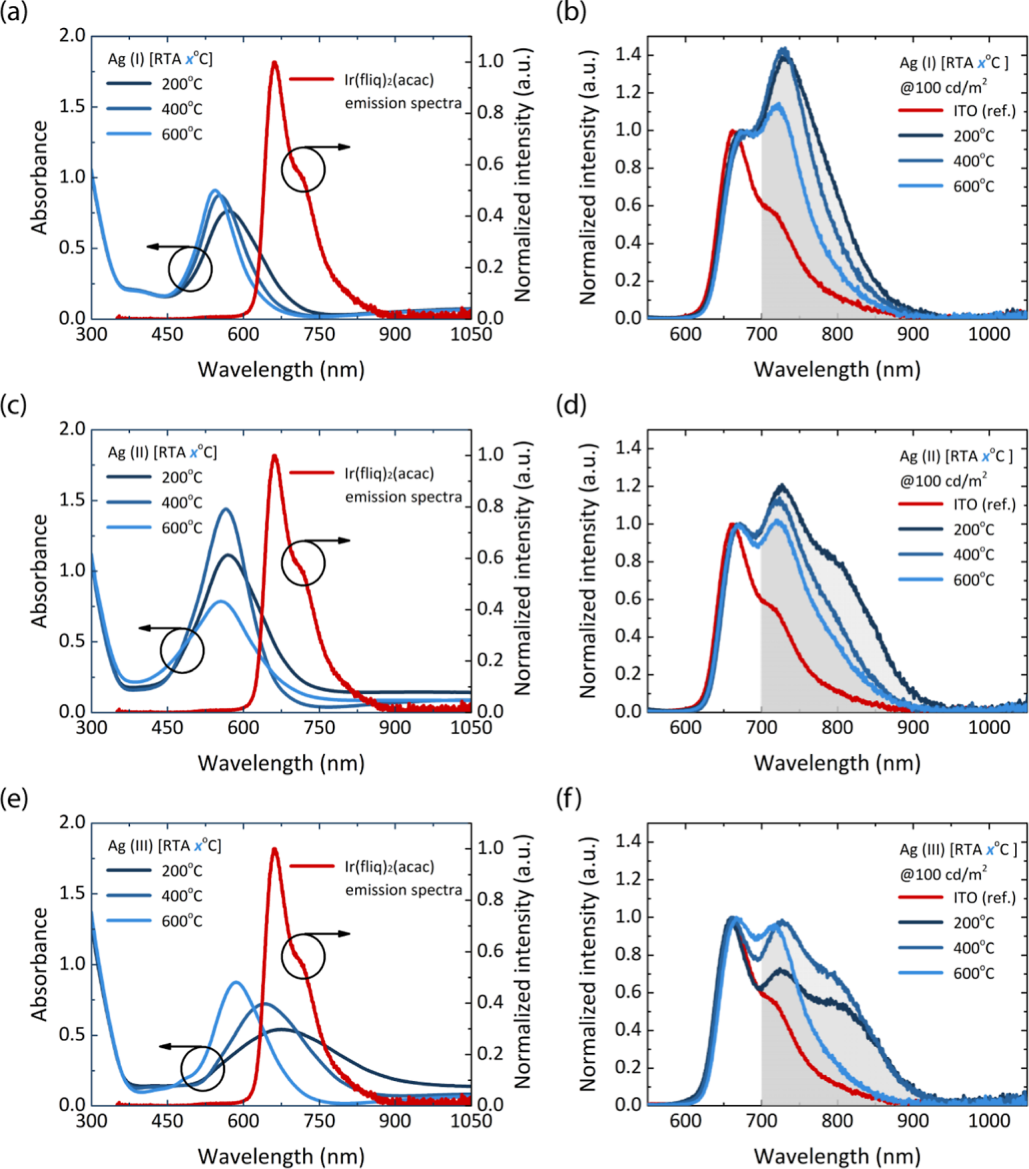
Thin film absorbance vs Ir­(fliq)_2_(acac) emission spectra and normalized EL spectrum of various TiO_2_ coating techniques under different RTA treatments for the devices (a,b) Ag (I), (c,d) Ag (II), and (e,f) Ag (III).

As shown in Figure 5, device Ag (I) exhibits a relatively small cross-sectional absorption area, covering a limited portion of the initial EL spectrum (600–730 nm). This result in the narrowest absorption profile among the three coating techniques. The most significant EL shift is observed from 663 nm (reference device) to 734 nm at an RTA of 200 °C. This shift is attributed to the narrow absorption cross-section, which effectively captures the main peak of the red emitter. At an RTA of 200 °C, device Ag (I) achieved a peak EL intensity of 1.4 au, compared to 1.2 au for device Ag (II). The broader absorption spectrum of device Ag (II) (stacking structure) covered the entire emitter spectrum, leading to a lower overall EL intensity but inducing the emergence of a third peak at 714 nm due to LSPR-induced modulation of the second emitter peak. Device Ag (III) (sandwiching) showed a more pronounced modulation of the secondary emission peak with a limited shift in the primary peak, which can be attributed to the strong absorption of the initial EL band. Among all configurations, device Ag (II) yielded the broadest EL spectrum, with a FWHM of 199 nm under 200 °C RTA, supporting its potential as a broadband NIR emitter. These findings suggest that the weaker absorption of the stacking technique across the initial EL spectrum favors peak shifting while broadening the EL spectrum.

In contrast, Au NP-based devices exhibited weaker spectral shifts due to their reduced LSPR absorption and limited variation across RTA treatments (Figure S5). While minor EL shifts were observed, they were less pronounced compared with Ag NP-based devices. The EL spectrum pattern generally aligns with the absorption profile, where the emergence of a third peak results from altering the emitter’s second peak. The most notable shift occurred at 734 nm, with the highest FWHM value of 189 nm recorded for device Au (II) at RTA 400 °C. Like the Ag NP, the stacked coating technique exhibits higher FWHM values. Overall, the extent of EL modulation in Au NP-based devices was consistent with the relatively weak and less tunable LSPR profiles.

Since LSPR formation significantly influences the EL spectra, it is crucial to identify the most effective coating for optimizing OLED performance. Figure 6 shows the *L*–*V*–*J* plot and EQE characteristics of Ag NP-based devices, while their current and power efficiency are presented in Figure S7. The numeric data of the EL characteristics are summarized in Table 2. The reference ITO data in the table correspond to our red phosphorescent OLEDs using a pure ITO anode with a thickness of 130 nm. Despite the similar electrical properties among the different TiO_2_ coating strategies, the EL performance varied significantly. All Ag NP-based devices showed reduced turn-on voltages (*V*_on_) with increasing RTA, approaching the reference ITO value of 2.42 V. The best performing device, Ag (I) at 600 °C RTA, achieved an EQE of 12.91%, with respective current and power efficiencies of 1.19 cd/A and 1.53 lm/W—comparable to the ITO reference device (13.73%, 2.68 cd/A, 3.44 lm/W). The performance of the device with various TiO_2_ coating techniques progressively improves with the RTA treatments due to well-defined and narrow absorbance spectra, which enhance light absorption and provide high transmittance in the NIR region.

**Figure 6:**
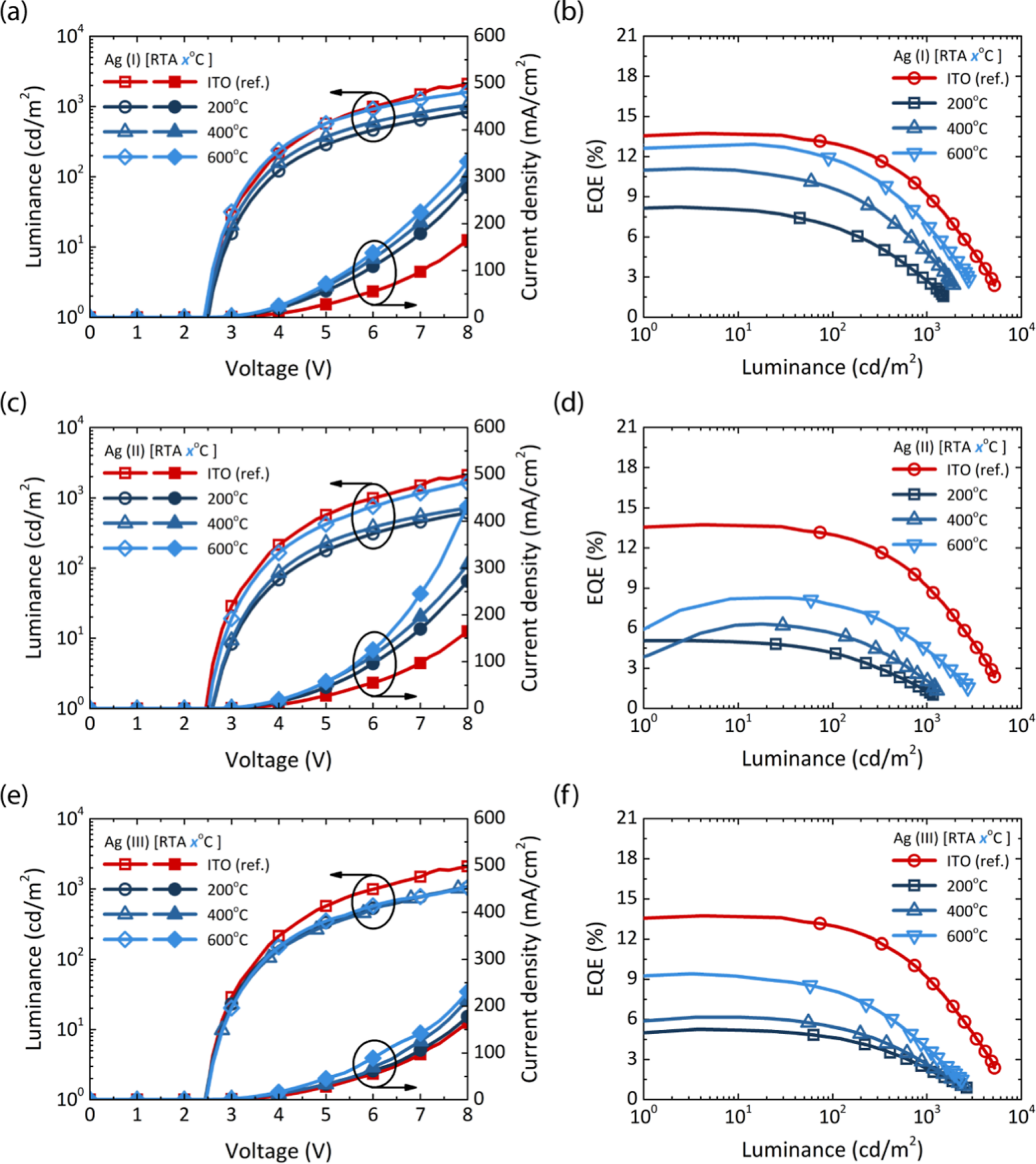
Electroluminescence performance summary, presented through the luminance–voltage–current density (*L*–*V*–*J*) plot and (d) EQE characteristics of various TiO_2_ coating techniques under different RTA treatments for the devices (a,b) Ag (I), (c,d) Ag (II), and (e,f) Ag (III).

**Table 2:** EL Spectrum and Performance Characteristics of Various TiO_2_ Coating Techniques under Different RTA Treatments for the Devices Ag (I), Ag (II), and Ag (III)

		TiO_2_ coating techniques								
device	ref. ITO	overcoating Ag (I)			stacking Ag (II)			sandwiching Ag (III)		
Ag NPs RTA [*x* °C]		200	400	600	200	400	600	200	400	600
first peak [nm]	663	**734**	731	723	727	726	719	660	663	669
second peak [nm]	714	672	688	681	667	675	669	724	727	717
FWHM [nm]	95	170	147	122	**199**	152	134	180	191	112
*V*_on_ [V]^a^	2.42	2.46	2.44	2.41	2.59	2.57	2.46	2.42	2.43	2.43
η_ext max_ [%]^b^	13.73	8.22	11.10	12.91	5.06	6.31	8.26	5.26	6.17	9.42
η_c max_ [cd/A]^c^	2.68	0.78	0.83	1.19	0.54	0.53	0.99	1.40	1.24	1.14
η_p max_ [lm/W]^d^	3.44	1.00	1.07	1.53	0.69	0.55	1.10	1.71	1.52	1.46
*L*_max_ [cd/m^2^]^e^	2090	831	1044	1604	608	719	1638	1051	1078	1046
CIE^f^	0.68, 0.30	0.67, 0.32	0.69, 0.30	0.70, 0.29	0.65, 0.33	0.67, 0.30	0.68, 0.31	0.64, 0.35	0.65, 0.34	0.70, 0.30

a Turn on voltage at 1 cd/m^2^.

b Maximum EQE.

c Maximum current efficiency.

d Maximum power efficiency.

e Maximum luminance at 8 V.

f CIE 1931 coordinate at 100 cd/m^2^.

For the Au NP-based devices (Figures S6 and S8; Table S3), EL performance was generally lower than that of the ITO reference and Ag NP-based devices. RTA and coating variations resulted in only marginal improvements, reinforcing the limited impact of Au NP LSPR on the device performance. This outcome is expected given that spectral modulation is strongly governed by the underlying absorption profile of each configuration. The Au NP LSPR profile is lower than that of the Ag NPs. Similar to the Ag NP, the Au NP-based devices provide notable EL performance with the overcoating technique of device Au (I) at RTA 600 °C, respectively, achieving a maximum EQE, CE, and PE of 8.74%, 1.24 cd/A, and 1.25 lm/W. However, LSPR formation using Au NPs apparently induces charge trapping rather than contributing to the OLED performance. This interpretation is supported by the higher mobility range observed for Au NP films (9.4–24.5 cm^2^ V^–1^ s^–1^) compared to Ag NP films (5–19.5 cm^2^ V^–1^ s^–1^), as seen in Tables 1 and S2. Additionally, while the current density of Ag NP-based devices significantly increases with annealing temperature, it decreases for Au NP-based devices, suggesting inefficient charge utilization in devices incorporating Au NPs. Although the Schottky junction between metallic NPs and TiO_2_ is known to facilitate interfacial charge trapping, particularly in Au/TiO_2_ systems,^67–69^ our observations regarding Ag and Au NPs under RTA treatment require further investigation to elucidate the underlying mechanisms.

The stacking technique results demonstrated an impressive FWHM value and successfully red-shifted the emitter’s main peak (660 nm) to the NIR region (>700 nm); thus, we further investigated the combination of Ag and Au NPs to explore hybrid effects. Two stacking-type configurations were tested: Ag–Au (Ag/TiO_2_/ITO/Au/TiO_2_) and Au–Ag (Au/TiO_2_/ITO/Ag/TiO_2_). The optical transmittance and absorbance profiles of these combinations under different RTA treatments are shown in Figure S9. Unfortunately, these thin film hybrid structures failed to outperform pure Ag systems. NIR transmittance remained below 80%, and the absorption intensity did not improve significantly with minimal variation across RTA treatment. These findings suggest that the inclusion of Au NPs disrupts the LSPR activity of the Ag NPs. The presence of Au also introduces additional plasmon resonance damping, resulting in broadened, shifted, or reduced LSPR peaks compared to those observed in pure Ag NPs.^70^ This damping effect is attributed to the interband transition of Au near 2.3 eV, which lies within the visible light spectrum. This transition allows Au to absorb part of the incident light and compete with the LSPR excitation of Ag. As a result, the presence of Au reduces the effective absorption of Ag and damps the overall plasmonic resonance.^71^

## 3 Conclusions

This study demonstrates a practical approach to achieving spectral reshaping and red-shifting in red phosphorescent OLEDs by leveraging LSPR induced by metallic NP-based anodes. Ag and Au NPs were integrated with TiO_2_ coatings using three distinct configurations—overcoating, stacking, and sandwiching—and subjected to RTA treatments at 200 °C, 400 °C, and 600 °C. SEM and UV–vis analyses confirmed that higher annealing temperatures and TiO_2_ coatings enhanced NP island formation, reduced agglomeration, and produced well-defined narrow LSPR absorbance peaks. Ag NP-based anodes exhibited significantly stronger LSPR responses than Au, resulting in more pronounced redshifts in EL spectra. The best device performance was achieved by the overcoating configuration (Ag (I)) under 600 °C RTA, with a maximum EQE of 12.91%, a current efficiency of 1.19 cd/A, and a power efficiency of 1.53 lm/W, closely matching the reference ITO device (13.73%, 2.68 cd/A, and 3.44 lm/W). The stacked coating technique produces higher FWHM values, with a maximum of 199 nm for device Ag (II) at RTA 200 °C and 189 nm for device Au (II) at RTA 400 °C. These findings suggest that LSPR formation with weaker absorption across the initial EL spectrum favors peak shifting while broadening the EL spectrum. In contrast, Au NP-based devices contributed minimally to EL spectral modulation and efficiency enhancement, likely due to weaker LSPR and possible charge-trapping effects. Hybrid Ag–Au or Au–Ag thin film structures failed to outperform pure Ag configurations, underscoring the superior plasmonic activity of Ag NPs. Overall, this work offers a practical path toward color-tunable NIR OLEDs, with promising implications for multifunctional optoelectronic and wearable photonic applications.

## 4 Experimental Section

Anode preparation and device fabrication: All materials utilized in this study were purchased from Shine Material Technology, including 1,4,5,8,9,11-hexaazatriphenylene hexacarbonitrile (HAT-CN), 1,1-bis­[(di-4-tolylamino)­phenyl]­cyclohexane (TAPC), 4,4′,4″-tris­(carbazol-9-yl)­triphenylamine (TCTA), bis­[1-(9,9-dimethyl-9*H*-fluoren-2-yl)-isoquinoline]­(acetylacetonate)­iridum­(III) (Ir­(fliq)_2_(acac)), 3′,3‴,3′″″-(1,3,5-triazine-2,4,6-triyl)­tris­(([1,1′-biphenyl]-3-carbonitrile)) (CN-T2T), and LiF. These materials were used without further purification. A cleaning procedure was conducted using ultrasonication of the ITO-coated glass in deionized water and organic solvents. The transparent anode structures, such as Ag NPs, titanium oxide (TiO_2_), and ITO, were prepared by using a sputtering machine (Co-Sputter System, Kao Duen Technology Corporation). High-purity sputtering targets of Ag (99.99%), TiO_2_ (99.99%), and ITO (99.99%) were purchased from Gredmann Taiwan Ltd. The chamber was evacuated to <4 × 10^–6^ Torr before introducing high-purity argon (Ar) gas. The sputtering condition of Ag was maintained at 2 mTorr and 20 sccm Ar flow with a 50 W DC power (SDC 1022A, PSPower). TiO_2_ and ITO were deposited at 1 mTorr and 30 sccm Ar flow using RF power at 100 and 50 W, respectively (CESAR RF Power Generator, Advanced Energy). Due to limitations, the Au NPs were fabricated using thermal evaporation in a vacuum chamber. The RTA was done using an RTA machine (eRTA 100, Giant Tek Company). After the ITO deposition process, the substrate was exposed by using an atmospheric pressure plasma treatment machine (SAP009SA, Creating Nano Technology Inc.) to improve the surface work function. The OLED device fabrication and Au NPs thin layer film were fabricated through thermal evaporation in a vacuum chamber maintained at a low pressure of approximately ∼10^–6^ Torr. The OLED deposition process was completed in a single cycle without interrupting the vacuum environment. Deposition rates were controlled within 1 Å s^–1^ for organic materials, while 5 Å s^–1^ was used for metal materials. Devices were encapsulated immediately using UV-curable epoxy and cover glass inside a nitrogen-filled glovebox (O_2_ and H_2_O < 0.5 ppm).

Device characterization: The optical properties of Ag and Au NPs, such as transmittance and absorbance, were measured by using a UV–vis spectrophotometer (Shimadzu UV-1650PC). The Ag and Au NP’s surface morphology, particle size, and NP distribution in the thin films were measured using SEM (JEOL JSM-7610FPlus). The film sample was coated using a platinum (Pt) conductive layer to improve the sample image using the auto fine coater machine (JEOL JEC-3000FC). EL spectra, current–voltage (*J*–*V*) characteristics, and EQE measurements of OLEDs were conducted at room temperature using a source meter (Keysight B2901A) and a fiber-coupled UV–vis spectrometer (Ocean Optics USB4000). Regarding error limits, measurement uncertainties based on instrument specifications are as follows: wavelength accuracy and reproducibility of ±0.5 and ±0.1 nm, respectively, for the spectrophotometer (Shimadzu UV-1650PC); spectral resolution of ±1.5 nm for the spectrometer (Ocean Optics USB4000); and a minimum source and measurement capability of 1 pA/1 μV and 100 fA/100 nV, respectively, for the source meter (Keysight B2901A).

## Supplementary Material



## Notes

Optical transmittance and absorbance of the various metallic NPs and different thicknesses of Ag NPs; thin film layer structure, optical transmittance, and absorbance of various TiO_2_ coating technique of Au NPs; absorbance vs Ir­(fliq)_2_(acac) emission spectra, normalized EL spectrum, luminance–voltage–current density (*L*–*V*–*J*), EQE characteristics, current and power efficiency of various TiO_2_ coating technique of Au NPs; current and power efficiency of various TiO_2_ coating technique of Ag NPs; thin film layer structure, optical transmittance, and absorbance for stacking coating technique using different combinations between Ag and Au NPs; sheet resistance of various metallic NP under different deposition thicknesses; summarized electrical properties and EL spectrum and performance characteristics of various TiO_2_ coating technique of Au NPs (PDF)
