# Supplementary material for: Harnessing Metallic Nanoparticle-Based Anodes for Red-Shifting and Reshaping Electroluminescence toward the Near-Infrared Region
Source: ACS Appl Mater Interfaces. 2025 Jul 7;17(28):40881–92. doi: 10.1021/acsami.5c08869 (PMC12278203; doi:10.1021/acsami.5c08869)
Supplement: Supplementary file 1 [file am5c08869_si_001.pdf]

Supporting Information

Harnessing Metallic Nanoparticle-Based Anodes for Red-Shifting and Reshaping Electroluminescence toward the Near-Infrared Region

Nurul Ridho Al Amin,[a] *Ming-Jun Lin,*[a] *Jui-Ming Wang,*[a] *Zu-Po Yang,*[b] *Hai-Ching Su,*[c,*] *Chih-Hao Chang*[a,*]

[a]Department of Electrical Engineering, Yuan Ze University, Taoyuan, 32003, Taiwan.

[b]Institute of Photonic System, National Yang Ming Chiao Tung University, Tainan 71150, Taiwan.

[c]Institute of Lighting and Energy Photonics, National Yang Ming Chiao Tung University, Tainan, 71150, Taiwan.

[*]Corresponding authors. Email address: chc@saturn.yzu.edu.tw (Dr. Chih-Hao Chang)

haichingsu@nycu.edu.tw (Dr. Hai-Ching Su)

KEYWORDS: localized surface plasmon resonance, color-tunable, silver nanoparticles, gold nanoparticles, organic light-emitting diodes

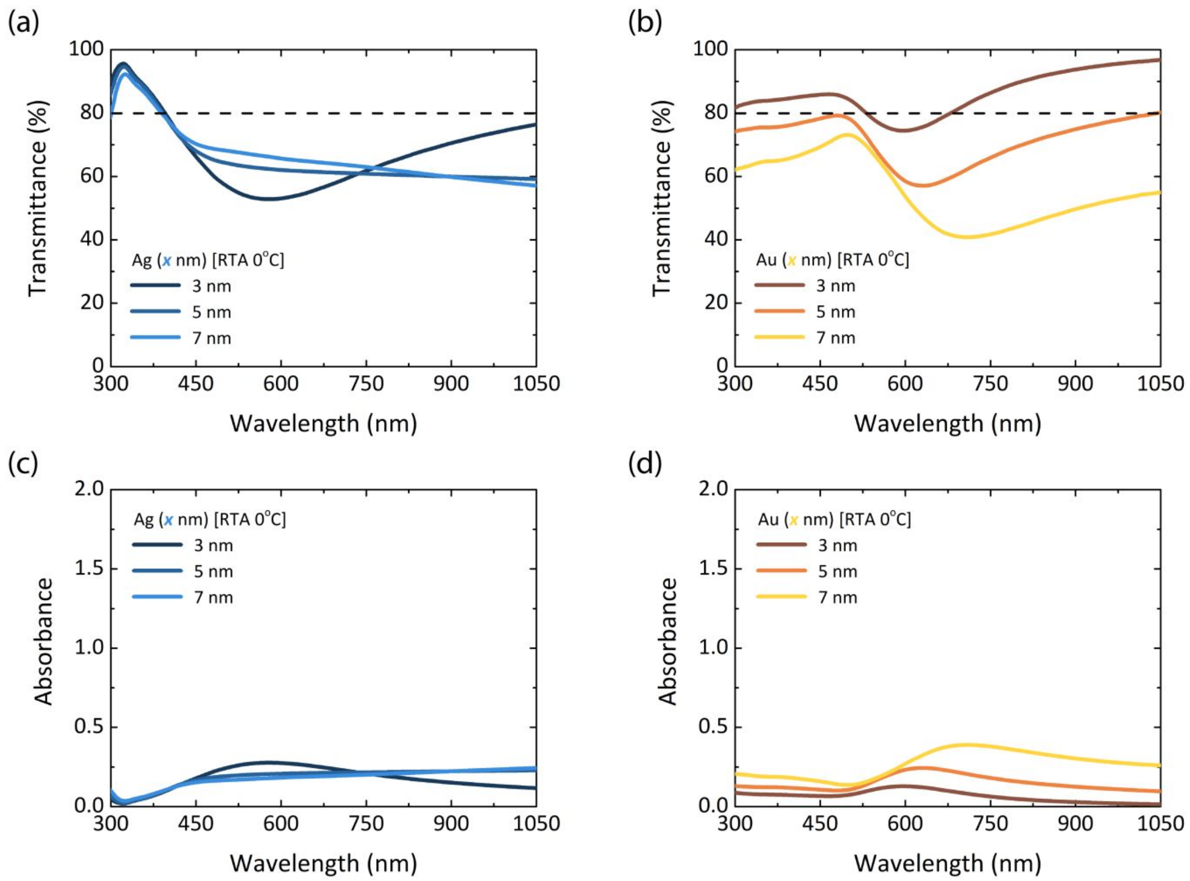

Figure S1. Optical transmittance and absorbance of various metallic nanoparticle thin films under different deposition thicknesses for (a), (c) Ag NPs, and (b), (d) Au NPs.

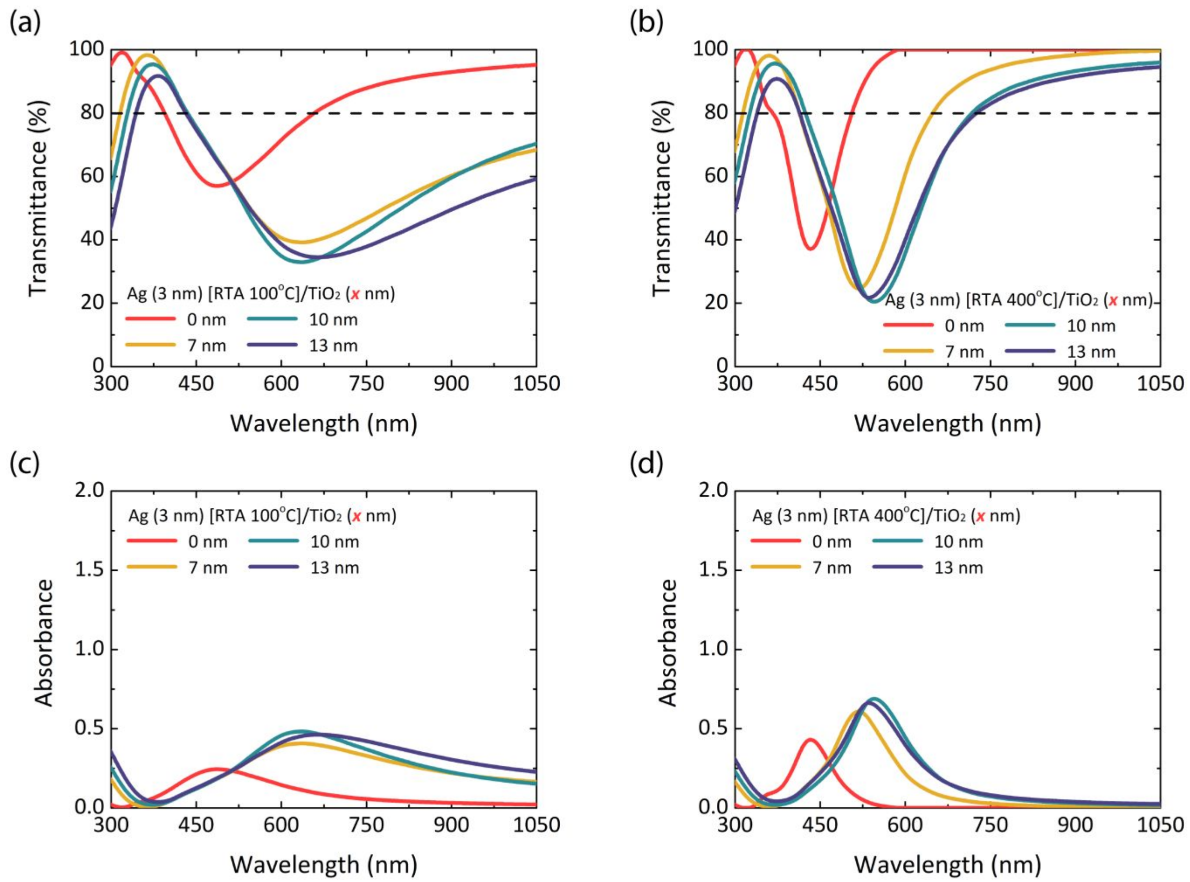

Figure S2. Optical transmittance and absorbance of the Ag nanoparticle thin films under different deposition thicknesses for (a), (c) Ag NPs (RTA 100°C), and (b), (d) Ag NPs (RTA 400°C).

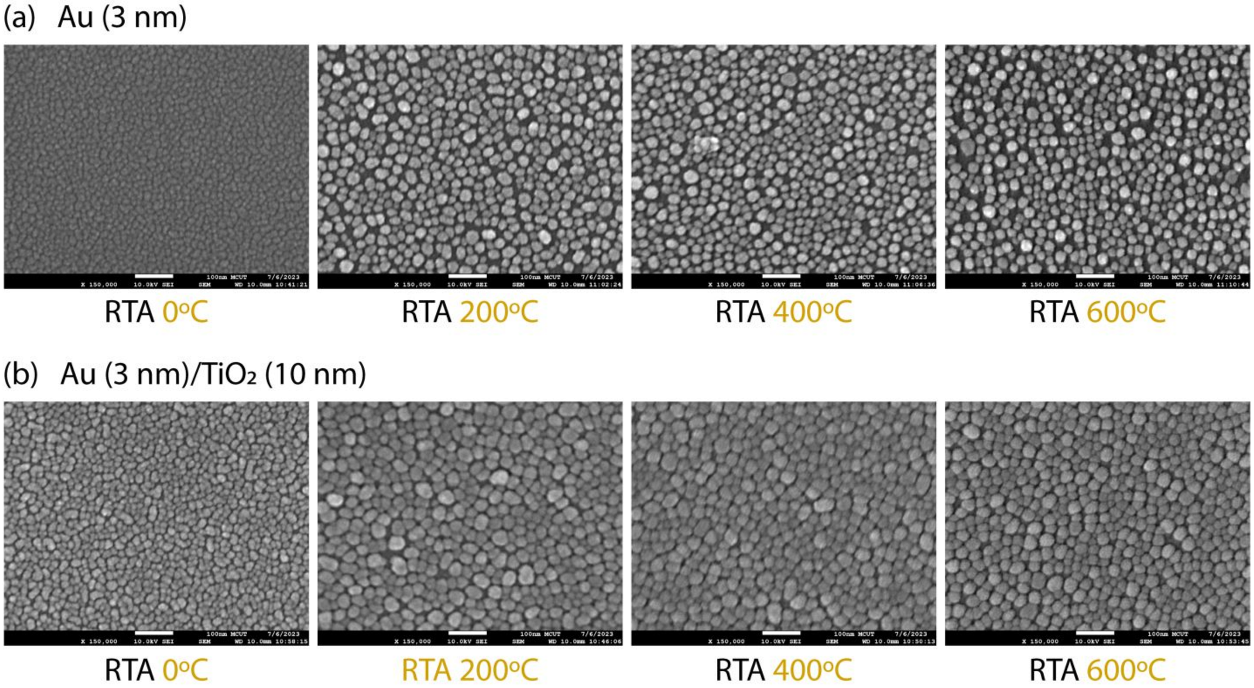

Figure S3. SEM images of Au nanoparticle thin films under different RTA treatments for (a) Au and (b) Au/TiO_2.

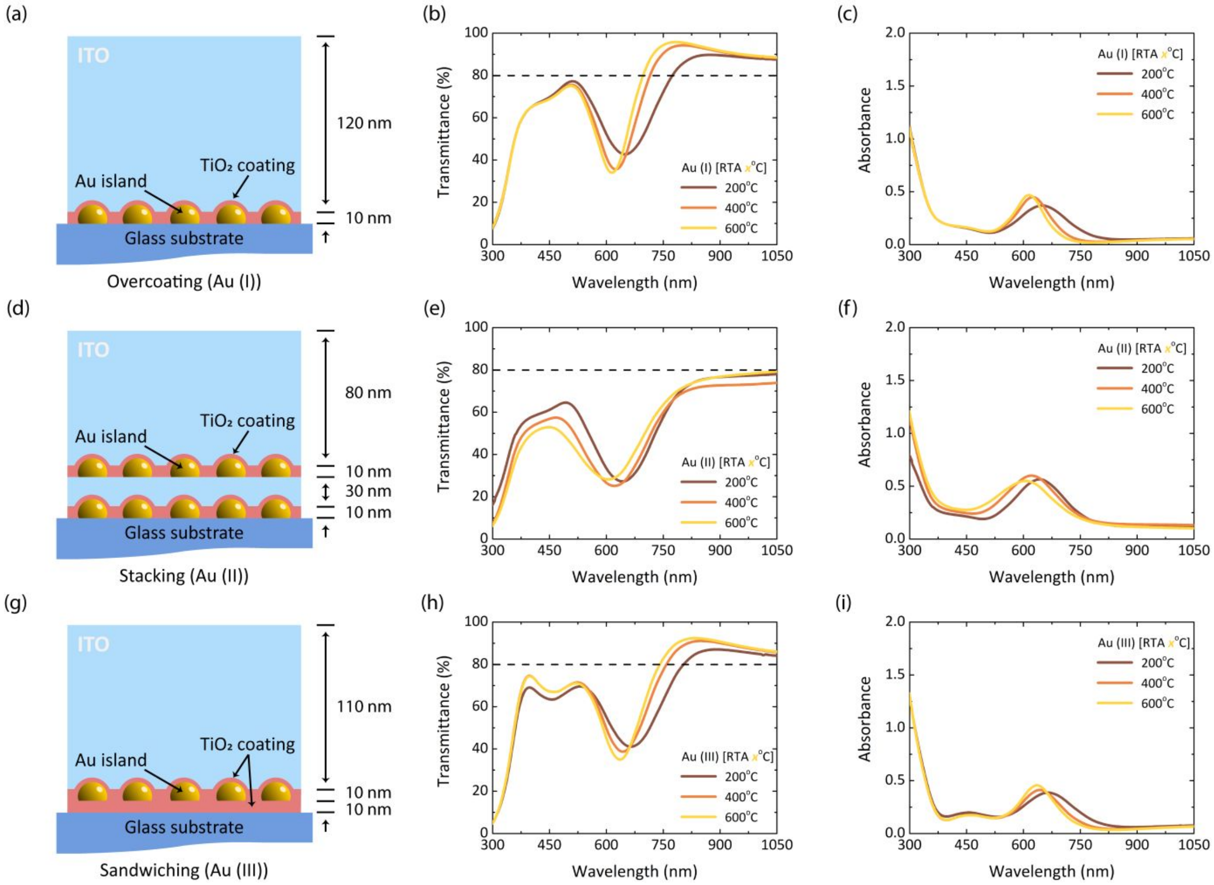

Figure S4. Thin film layers produced by various TiO_2 coating techniques using Au nanoparticles deposited on glass substrate for (a) Overcoating (Au (I)): Au/TiO_2, (d) Stacking (Au (II)): Au/TiO_2/ITO/Au/TiO_2, and (g) Sandwiching (Au (III)): TiO_2/Au/TiO_2. The optical transmittance and absorbance of various TiO_2 coating techniques under different RTA treatments for the thin films (b), (c) Au (I), (e), (f) Au (II), and (h), (i) Au (III).

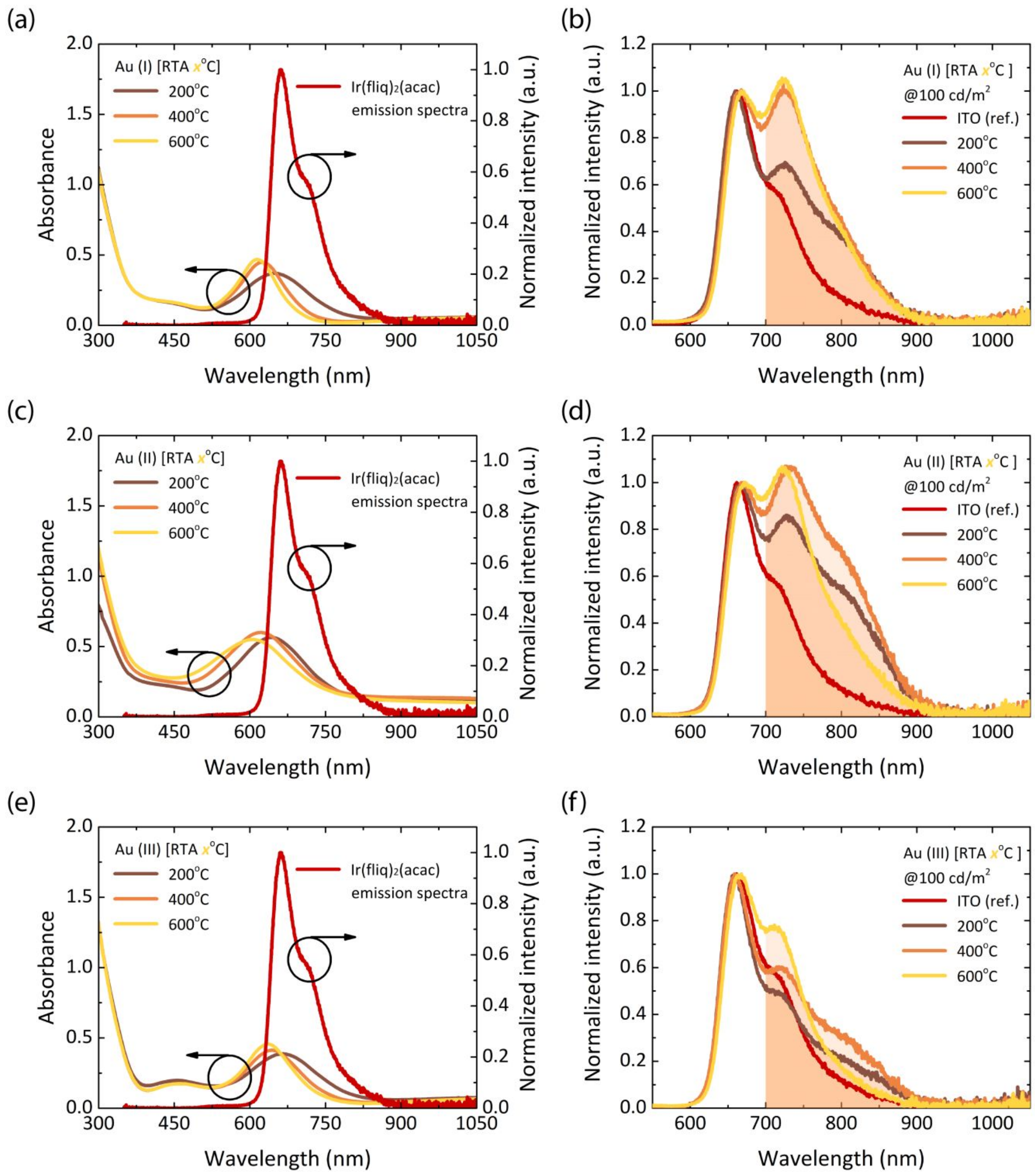

Figure S5. Thin film absorbance vs. Ir(fliq)$_2$(acac) emission spectra and normalized EL spectrum of various TiO_2 coating techniques under different RTA treatments for the devices (a), (b) Au (I), (c), (d) Au (II), and (e), (f) Au (III).

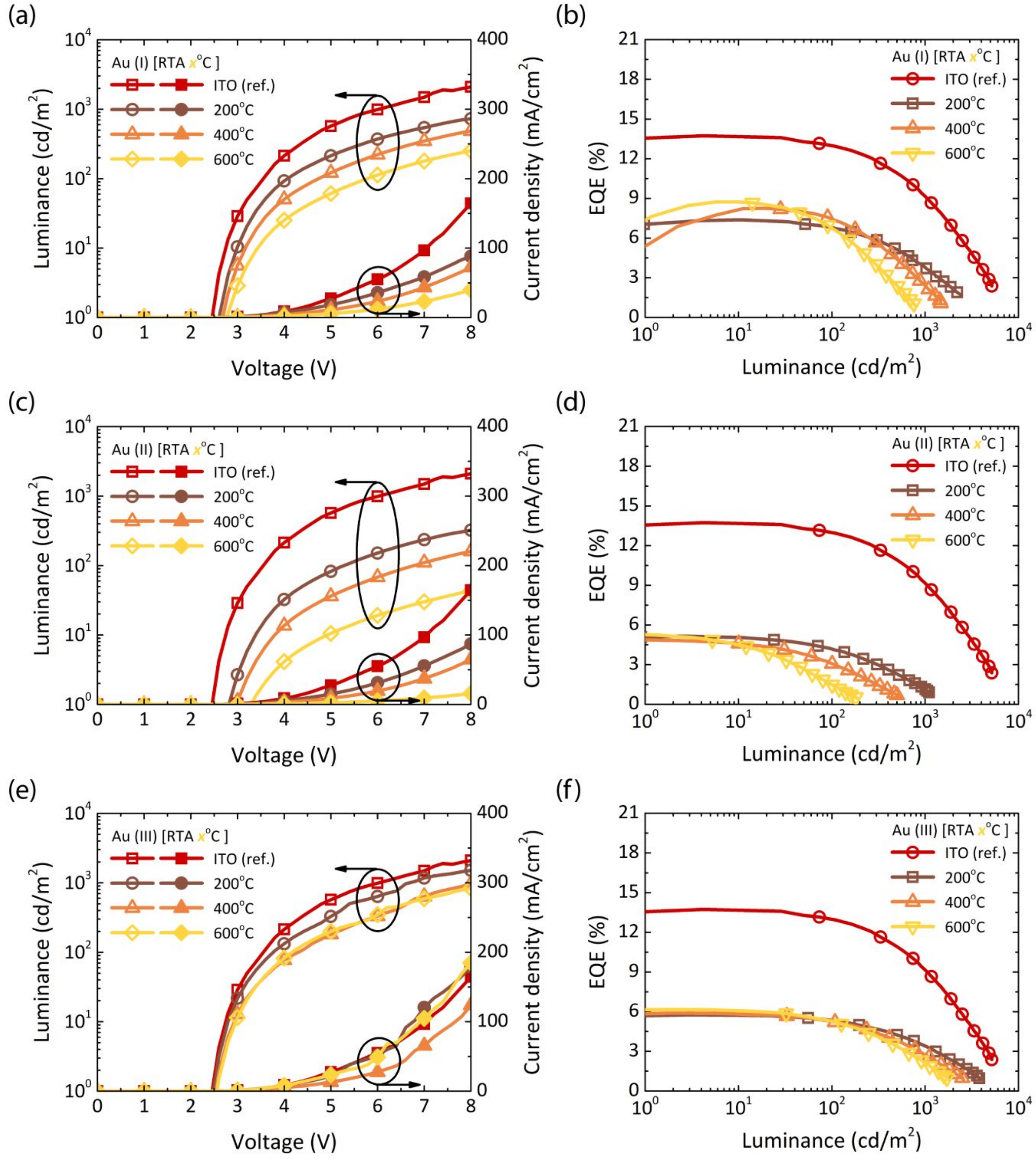

Figure S6. Electroluminescence performance summary, presented through the luminance-voltage -current density (*L-V-J*) plot and (d) EQE characteristics of various TiO_2 coating techniques under different RTA treatments for the devices (a), (b) Au (I), (c), (d) Au (II), and (e), (f) Au (III).

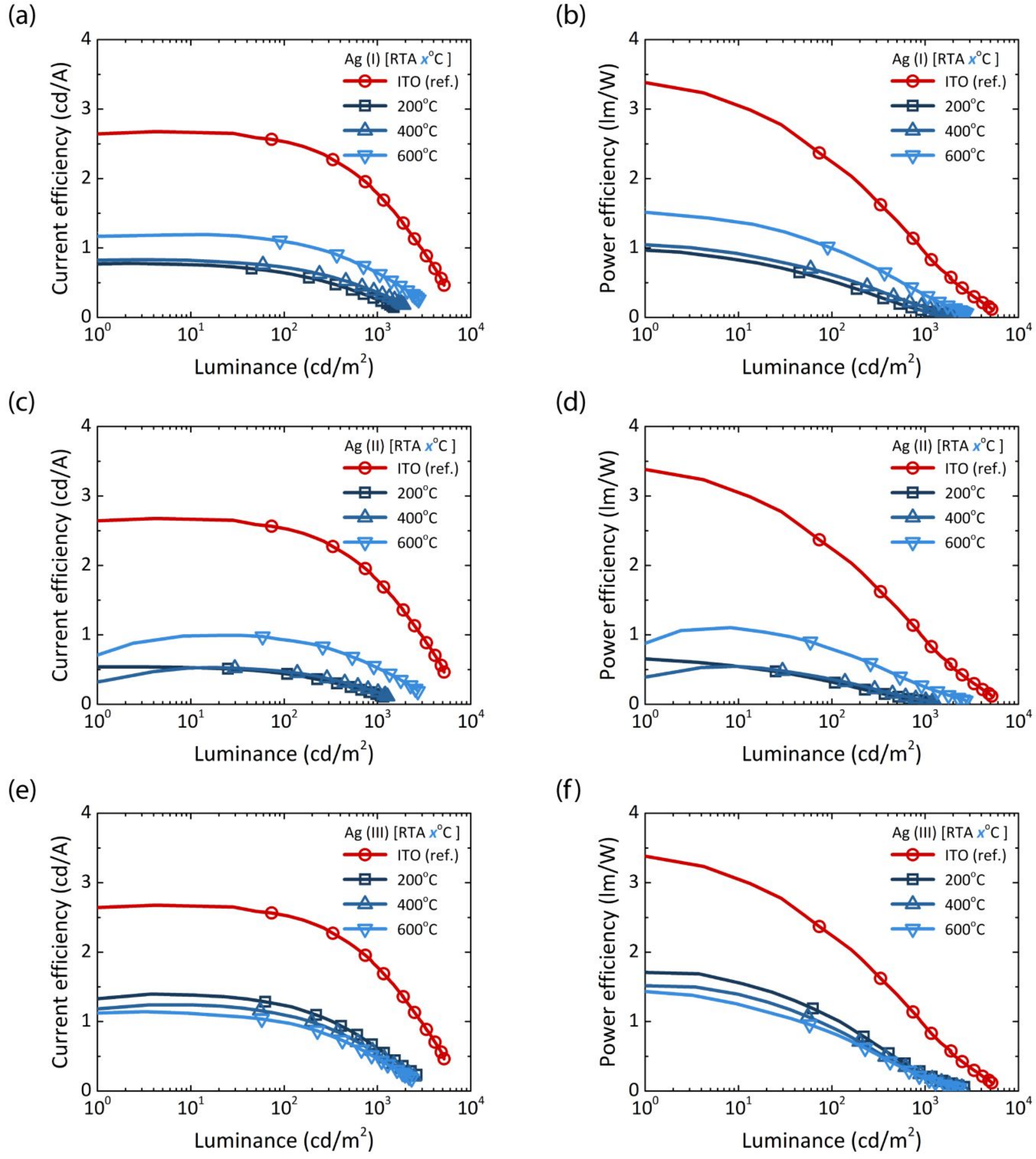

Figure S7. Current and power efficiencies produced by various TiO_2 coating techniques under different RTA treatments for the devices (a), (b) Ag (I), (c), (d) Ag (II), and (e), (f) Ag (III).

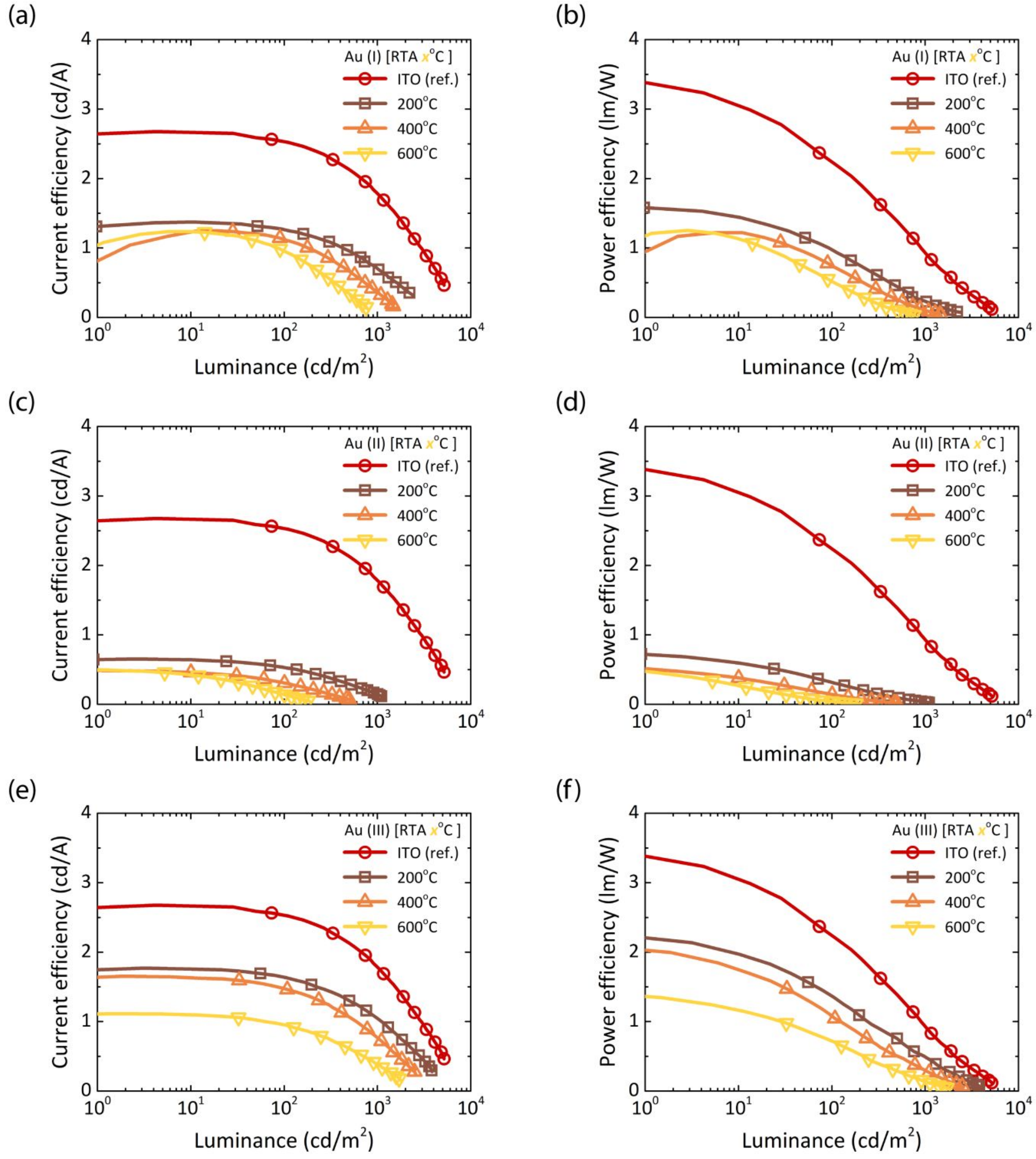

Figure S8. Current and power efficiency produced by various TiO_2 coating techniques under different RTA treatments for the devices (a), (b) Au (I), (c), (d) Au (II), and (e), (f) Au (III).

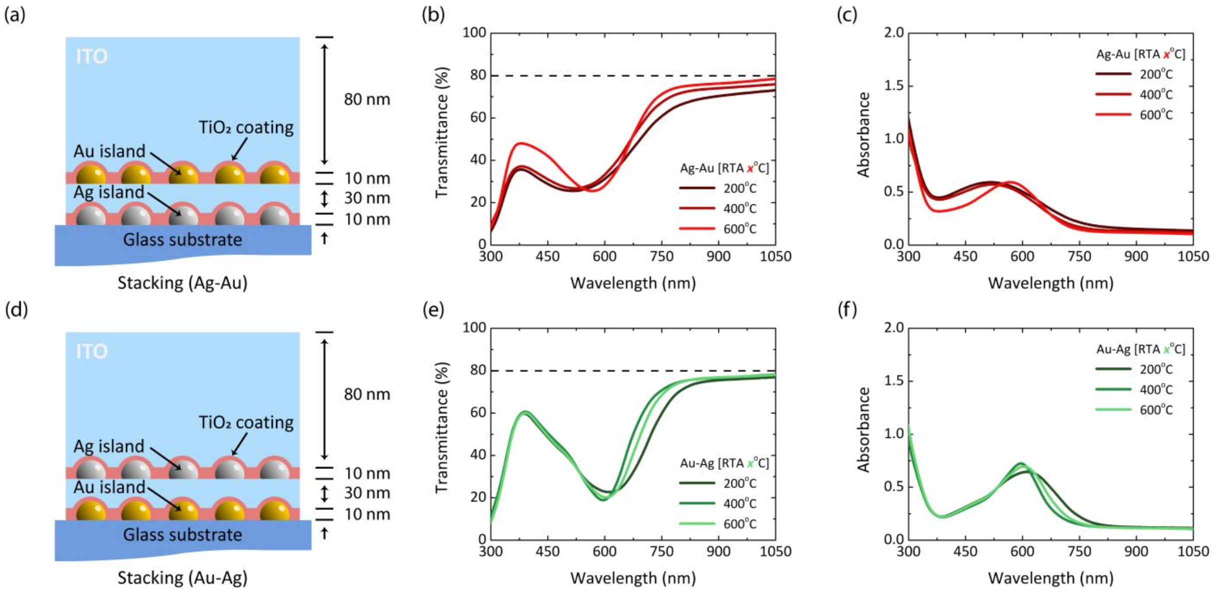

Figure S9. Thin film layer of stacking TiO_2 coating techniques using different combinations of silver and gold nanoparticles deposited on a glass substrate for (a) Ag-Au: Ag/TiO_2/ITO/Au/TiO_2, and (d) Au-Ag: Au/TiO_2/ITO/Ag/TiO_2. The optical transmittance and absorbance of stacking TiO_2 coating techniques under different RTA treatments for the thin films (b), (c) Ag-Au, and (e), (f) Au-Ag.

Table S1. Sheet resistance of various metallic nanoparticle thin films on top of a glass substrate under different deposition thicknesses.

**Material**	**Ag NPs**			**Au NPs**		
Thickness [nm]	3	5	7	3	5	7
Rs [Ω sq^{-1}]	7.5×10^8	4085	22	1.3×10^8	1586	15

Table S2. Electrical properties of the Au nanoparticle-based anode using various TiO_2 coating techniques under different RTA treatments for the thin films Au (I), Au (II), and Au (III).

**Thin film**	**TiO_2 coating techniques**								
	**Overcoating Au (I)**			**Stacking Au (II)**			**Sandwiching Au (III)**		
Structure	Glass/**Au (3 nm)**/TiO_2 (10 nm)/ITO (120 nm)			Glass/**Au (3 nm)**/TiO_2 (10 nm)/ITO (30 nm)/**Au (3 nm)**/TiO_2 (10 nm)/ITO (80 nm)			Glass/TiO_2 (10 nm)/**Au (3 nm)**/TiO_2 (10 nm)/ITO (110 nm)		
Au NPs RTA [***x***°C]	200	400	600	200	400	600	200	400	600
**n** [$\times 10^{21}$ cm^{-3}]	2.5	2.7	1.4	1.4	1.6	1.5	1.1	0.7	0.6
**μ** [cm^2 V^{-1} s^{-1}]	15.7	15.3	9.4	17.7	15.0	17.5	14.2	20.1	24.5
**ρ** [$\times 10^{-4}$ Ω cm]	4.9	4.9	5.0	4.8	3.7	3.2	4.6	4.8	4.8
**Rs** [Ω sq^{-1}]	37.7	37.8	38.2	36.5	28.3	24.6	35.6	37.1	37.2

n: carrier concentration, *μ*: mobility, *ρ*: resistivity, *Rs*: sheet resistance.

Table S3. EL spectrum and performance characteristics of various TiO_2 coating techniques under different RTA treatments for the devices Au (I), Au (II), and Au (III).

**Device**	**Ref. ITO**	**TiO_2 coating techniques**								
		**Overcoating Au (I)**			**Stacking Au (II)**			**Sandwiching Au (III)**		
Au NPs RTA [***X***°C]	–	200	400	600	200	400	600	200	400	600
Main peak [nm]	663	660	723	722	668	**734**	721	659	659	668
2nd peak [nm]	714	726	667	668	727	668	672	709	718	712
FWHM [nm]	95	125	148	142	174	**189**	139	73	105	104
V_{on} [V][a]	2.42	2.60	2.65	2.76	2.81	2.97	3.27	2.44	2.49	2.52
$\eta_{ext\ max}$ [%][b]	13.73	7.38	8.26	8.74	5.15	4.87	5.32	5.77	5.89	6.16
$\eta_{c\ max}$ [cd/A][c]	2.68	1.37	1.25	1.24	0.65	0.49	0.50	1.77	1.65	1.11
$\eta_{p\ max}$ [lm/W][d]	3.44	1.58	1.22	1.25	0.73	0.55	0.52	2.26	2.10	1.43
L_{max} [cd/m^2][e]	2089.78	729.75	488.21	252.57	323.70	161.00	43.10	1513.42	968.75	831.68
CIE[f]	0.68, 0.30	0.67, 0.31	0.67, 0.32	0.66, 0.33	0.69, 0.30	0.68, 0.31	0.67, 0.31	0.66, 0.33	0.65, 0.33	0.68, 0.31

[a] turn on voltage at 1 cd/m^2, [b] maximum EQE, [c] maximum current efficiency, [d] maximum power efficiency, [e] maximum luminance at 8 V, and [f] CIE 1931 coordinate at 100 cd/m^2
